# Ultra-low-power switching circuits based on a binary pattern generator with spiking neurons

**DOI:** 10.1038/s41598-022-04982-w

**Published:** 2022-01-21

**Authors:** Takeaki Yajima

**Affiliations:** Department of Electronics, Kyushu University, Fukuoka-shi, Fukuoka 819-0395 Japan

**Keywords:** Electrical and electronic engineering, Devices for energy harvesting

## Abstract

Research on various neuro-inspired technologies has received much attention. However, while higher-order neural functions such as recognition have been emphasized, the fundamental properties of neural circuits as advanced control systems have not been fully exploited. Here, we applied the functions of central pattern generators, biological neural circuits for motor control, to the control technology of switching circuits for extremely power-saving terminal edge devices. By simply applying a binary waveform with an arbitrary temporal pattern to the transistor gate, low-power and real-time switching control can be achieved. This binary pattern generator consists of a specially designed spiking neuron circuit that generates spikes after a pre-programmed wait time in the six-order range, but consumes negligible power, with an experimental record of 1.2 pW per neuron. This control scheme has been successfully applied to voltage conversion circuits consuming only a few nanowatts, providing an ultra-low power technology for trillions of self-powered edge systems.

## Introduction

Due to the development of neuroscience and the success of machine learning technology, there has been increasing interest in neuro-inspired technologies that focus on the neural circuits from an engineering viewpoint. The interest is not limited to algorithmic research, but also extends to hardware research, which aims to implement neuro-inspired circuits and systems with lower power consumption and low latency^1–8^. However, most of the current hardware research targets the understanding of biological processes or the implementation of machine learning algorithm, and may not fully exploit the tremendous potential of biological neural circuits.

**Figure 1:**
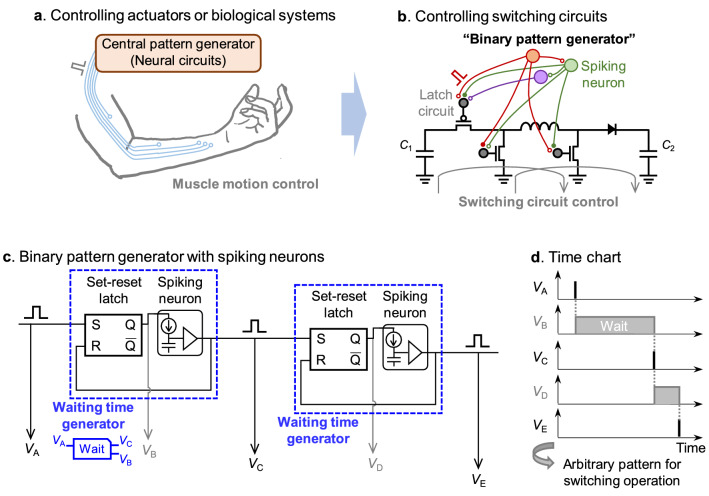
Binary pattern generator. (**a**) A central pattern generator in the human neural network which controls biological motors. (**b**) A concept of an artificial pattern generator which controls switching circuits for DC-DC voltage conversion. (**c**) A binary pattern generator, a specially designed pattern generator for purpose of controlling switching circuits. It consists of a chain of waiting time generators with a wide range of preprogrammed waiting times. (**d**) The spike or bit output signals from the binary pattern generator. The length of the bit signal corresponds to the preprogrammed waiting time of each waiting time generator. This bit signal is directly used for the gate control in the switching circuits.

In the engineering perspective, biological neural circuits are excellent control systems that control body movements with low power consumption and low latency. It compromises the trade-off between power consumption and response time by automating and decentralizing individual motor control, rather than fast centralized feedback control of body movements. There are two types of such decentralized motor control: one using reflexes that show a fixed response to a specific sensory input, and the other using a central pattern generator that drives motor organs according to a programmed time pattern^9,10^. While the reflex circuit is a relatively simple input–output system, the central pattern generator is a neuronal network that autonomously generates temporal patterns without input (Fig. 1a), and is responsible for complex and rhythmic motor control such as walking, chewing, breathing, and swallowing^11,12^. The central pattern generator generally uses spike signals that are heterogeneous with respect to time. This is essentially different from the clock signal in digital circuits that ticks at a fixed period independent of the environment. Compared with the temporally homogeneous clock signal, the spikes at heterogeneous timings can save redundancy, and hence, more suitable for real-time operation.

So far, several central pattern generators have been artificially fabricated in the form of electronic circuits for controlling robot actuators^13–17^, biological muscles^18^, and physiological systems^19^. However, there are few examples of their application to controlling more general electronic circuits, such as the switching circuits (Fig. 1b). The switching circuit consists of several transistor switches that are turned on and off rapidly to provide a fine-tuned averaged function. They are essential building blocks in various fields of electronics including the Internet of Things (IoT) devices. In parallel with the artificial central pattern generators for actuators and biological systems, the pattern generators for switching circuits have the potential to provide the real-time and energy-saving control scheme in the future IoT devices.

In this study, we constructed an artificial pattern generator that is optimized for switching circuits rather than for actuators or biological systems. The pattern generators in the previous studies often uses the spike rate coding, which convey analog information by spiking frequency. On the other hand, the switching circuits only needs timing information of the switching event, and therefore, it is more convenient to use temporal coding, which convey information as the timing of each spike. One of the essential components for temporal coding is the waiting time generator, which generates a spike signal after a preprogrammed waiting time from the onset of the input voltage. In addition, a wide range of waiting times are needed because the switching circuits sometimes change rapidly on the order of nanoseconds while waiting for most of the time to save energy. In this study, we experimentally demonstrated this wide range of waiting times from 100 ns to 100 ms over six orders of magnitude. In order to generate the arbitrary waiting time and an output spike signal, a technique of spiking neuron circuits with integrate-and-fire function was adopted^20–24^. The spiking neuron circuits were optimized solely for the purpose of waiting time generation based on the complementary metal oxide semiconductor (CMOS) technology, and any other biological function was not implemented intentionally. As a result, they achieved extremely low energy consumption in the order of 100 fJ per spike, corresponding to the average power consumption of 1.2 pW in minimum, which is the smallest ever among past experimental demonstrations^20–24^. For controlling the switching circuit, the input and the output of the above CMOS spiking neuron circuit were connected to a set-reset latch circuit to generate a binary wave form with a length of the preprogrammed waiting time as shown in Fig. 1c,d. Then, this binary wave form was simply applied to the transistor gates to control the switching circuits asynchronously. This simple scheme of the “binary pattern generator” can be applied to a versatile switching circuits and can achieve real time control in an extremely low power. For demonstration, we used this binary pattern generator to control the DC-DC voltage conversion circuit, which is essential for IoT devices, and showed by simulation that it can generate a wide range of output power between 8.36 nW and 1.16 mW at approximately 90% efficiency, with negligible control power consumption around three orders smaller than the output power. Thus, controlling switching circuits with a binary pattern generator provides a powerful means to alleviate the power constraints and realize various functions in self-powered IoT terminal devices.

In this paper, we first explain the detailed concept of the binary pattern generator, which is a specially designed pattern generator for controlling the switching circuits. Then, we visualize how it works in the demonstration of the switching circuit for DC-DC voltage conversion. Finally, the simulation and experiments are shown for the CMOS spiking neuron circuits that are the essential building blocks for the binary pattern generator.

### Binary pattern generator

Artificial central pattern generators have been previously studied in the field of robotics^13–17^. There, the information is conveyed as the analog value of the spike rate (spike rate coding), which can be used to control the actuators of robots. The power consumption is dominated by the driving power of the actuators, and there is little need to reduce the control power consumption to the extreme. The control time scale is also determined by the motion speed of the robot, and so there is little need for a large range of control time down to nano seconds. On the other hand, if the artificial pattern generator is to be used for the switching circuits, it requires extremely low power consumption and a wide range of time scales down to nano seconds. Therefore, for controlling switching circuits, a novel artificial pattern generator is needed to meet all these demands.

To address this issue, a binary pattern generator was constructed based on the control scheme of temporal coding. Previously, the similar binary waveforms were also exploited in some of the studies on robot control^13,14^. To construct a binary patter generator, we used CMOS spiking neuron circuits with the integrate-and-fire function, which can generate a spike after a preprogrammed waiting time from the onset of the input step voltage. The details will be discussed in the later sections. Each spiking neuron circuit is combined with a set-reset latch circuit, a simplest memory circuit, as shown in Fig. 1c,d. When the latch circuit is turned on by an input spike signal, the latch circuit generates the output voltage of 1 V. Then, this output voltage is applied to the subsequent spiking neuron circuit, resulting in the output of a spike signal after a preprogrammed waiting time. This output spike from the spiking neuron circuit resets the initial latch circuit and simultaneously turns on the latch circuit of the next stage. By repeating this operation, spike signals are generated at arbitrary time intervals (*V*_A_ , *V*_C_, and *V*_E_ in Fig. 1c,d), and a binary pattern can be generated from the output of the latch circuits (*V*_B_ and *V*_D_). This binary pattern can then be used to control transistor gates in the switching circuits. Here, a combination of a latch circuit and a spiking neuron circuit are referred to as a waiting time generator (dashed square in Fig. 1c). It should be noted the spiking neuron circuit in the binary pattern generator is directly connected to the set-reset latch circuit, and therefore, should be compatible with the CMOS logic circuits. Specifically, it should operate under the same 1 V power supply as the logic circuits, and it should output spikes with sufficiently short rise times and fall times on the scale of nano seconds. All these requirements are satisfied by a special design of CMOS spiking neuron circuits as shown later.

### Application to voltage converter circuit

**Figure 2:**
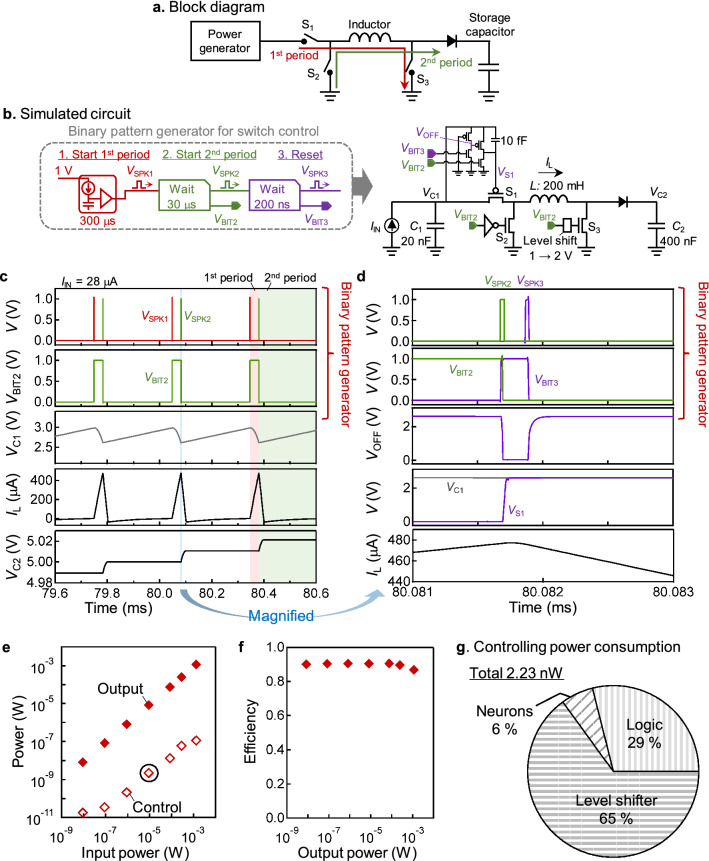
Application example of the binary pattern generator. (**a**) A block diagram of the DC-DC voltage converter circuit that consists of an inductor and several switches. (**b**) A circuit diagram of the simulated DC-DC voltage converter circuit, where the switching operation is controlled by the binary pattern generator. The binary pattern generator consists of a spiking neuron circuit defining the switching period, and two waiting time generators for switching sequences. The topology of the DC-DC voltage converter circuit is a typical buck-boost converter with a 200 mH inductor. (**c**) The voltage or current wave forms of the binary pattern generator and the DC-DC voltage converter circuit. (**d**) The wave forms of the binary pattern generator and the voltages around the S_1_ switch. The time span corresponds to the blue shadowed region in (c). (**e**) The simulated output power and the control power as a function of the input power when *V*_C1_ is around 3 V and *V*_C2_ is around 5 V. The input power is varied by changing the period of *V*_SPK1_ from 3 s to 50 μs. The control power is negligible, several orders smaller than the input or output power. (**f**) The simulated power conversion efficiency (the ratio of the output power to the input power) as a function of the output power, which remains a relatively high value down to an extremely low output power level. (**g**) The classification of the control power (2.23 nW) when the input power is 84 μW, which corresponds to a point with a solid circle in (**e**). The power consumption of the binary pattern generator (“Neurons”) is sufficiently small, even smaller than the logic circuit.

In order to visualize how the binary pattern generator works in the practical system, a simulation of the switching circuit for DC-DC voltage conversion is shown in Fig. 2. The DC-DC voltage conversion is an essential component for power supply circuits, especially for an energy harvesting IoT device^25^, where the time-varying generated power is buck-boost converted via an inductor and stored in a capacitor at a certain voltage level (Fig. 2a). The circuit operation consists of two periods: the first period in which the switches of S1 and S3 are turned on (red arrow), and the second period in which only the switch S2 is turned on (green arrow). In the first period, the charge stored in the primary capacitor (*C*_1_) flows to ground through S1 and S3, and the electrostatic energy of *C*_1_ is converted to magnetic flux energy of the inductor. In the second period, current flows from the ground to the secondary capacitor (*C*_2_) via S2, and the magnetic flux energy is converted into electrostatic energy of *C*_2_.

To control this switching operation, the binary voltages from the binary pattern generator were applied to the gate electrodes of the metal–oxide–semiconductor field effect transistors (MOSFETs) as shown in Fig. 2b. A binary pattern (*V*_BIT2_) was generated from the spike signals *V*_SPK1_ and *V*_SPK2_ as shown in Fig. 2c, and was used to control the gates of S1, S2 and S3. First, the *V*_SPK1_ is generated at a certain period, for example 300 μs, by the first-stage spiking neuron circuit, and the *V*_SPK2_ is generated 30 μs after *V*_SPK1_ by a waiting time generator. Then, the created binary pattern *V*_BIT2_ was inverted by an inverter and used for gate control of S2. At the same time, the voltage level of *V*_BIT2_ was raised from 1 to 2 V by a level shifter and used for gate control of S3. To control the gate voltage (*V*_S1_) of the S1, which is connected to the high voltage side in the circuit, a binary pattern (*V*_BIT3_) was generated from *V*_SPK2_ and an additional spike signal (*V*_SPK3_). When *V*_BIT3_ is turned on, *V*_OFF_ is pulled down, *V*_S1_ is pulled up, and S1 is turned off (Fig. 2d). On the other hand, when *V*_BIT2_ is turned on, *V*_S1_ is pulled down and S1 is turned on. In this way, the voltage level of the binary pattern is properly converted and used for gate driving, which makes the switching circuit extremely simple and low power.

When the input power of this circuit was varied from 9.28 nW to 1.33 mW by changing the period of *V*_SPK1_ from 3 s to 50 μs, the output power varied almost proportionally from 8.36 nW to 1.16 mW (Fig. 2e), and overall, the efficiency was around 90% (Fig. 2f). The comparison with the previous studies in Supplementary Note 1 shows the converter with a binary pattern generator maintains a higher efficiency than the previous ones without degradation down to 8.36 nW output power. The high efficiency at low output power is mainly because the power consumption of the control circuit was 2–4 orders of magnitude smaller than the output power throughout the range, for example, 17.5 pW for the output power of 8.36 nW, and 114 nW for the output power of 1.16 mW (Fig. 2e). In actual IoT devices, various control circuits need to be implemented, but as long as they can be controlled by the binary pattern generator, there may be no need to worry about the overhead of the control power even for an extremely low output power.

In order to clarify the cause of the low control power consumption, we examined the origins of the total control power of 2.23 nW when the input power was 84.4 μW (a point with a solid circle in Fig. 2e). The results in Fig. 2g show that the binary pattern generator operates at even lower power than the logic circuit, and this allows the control circuit as a whole to achieve very low power consumption. It should be noted the control power corresponds to the whole switching circuits but does not contains the power for the voltage detection on either side of the input or the output. This result suggests the possibility of the next-generation ultra-low power electronics, in which various functions can be implemented even with a very limited power of less than 1 μW if the control is based on a binary pattern generator.

### Simulation of spiking neuron circuit

**Figure 3:**
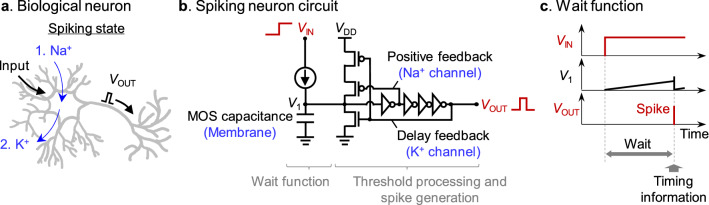
CMOS spiking neuron circuit. (**a**) A schematic illustration of a biological neuron. (**b**) A CMOS spiking neuron circuit with the integrate-fire function, which is used for the waiting time generator. (**c**) A schematic illustration of *V*_IN_, *V*_1_, and *V*_OUT_ in (**b**) as a function of time. A waiting time corresponds to the period between the onset of *V*_IN_ and the output of *V*_OUT_. The details of all the spiking neuron circuits with different waiting times are presented in Supplementary Note 3.

The most essential building blocks for the binary pattern generator are the waiting time generators which consist of spiking neuron circuits. So far, various neuron circuits have been created with different purposes in reference to biological neurons. In biological neurons, the membrane potential increases with each input, and when the threshold potential is reached, the influx of Na ions and the efflux of K ions alternate^26^, generating a spiking potential (Fig. 3a). This is accurately described by the Hodgkin–Huxley equation^27^, but a more simplified model is used in the design of neuron circuits^28^. In previous studies, relatively accurate analogue neuron circuits have been fabricated that implement the exponential behavior of ion channels in the subthreshold region of the transistor^29,30^. On the other hand, a further simplification of functions has been carried out in order to achieve large scale systems that includes a number of neuron circuits. Such simplified models include a integrate-fire neuron which is the simplest version^31^, a leaky-integrate-fire neuron which ignores variation of neuron dynamics^32^, an Izhikevich neuron which treats the firing process algorithmically^33^, and a phase transition neuron which implements functions with material properties^34,35^. In these analogue neuron circuits, there is a trade-off between accuracy and simplicity, but by focusing on the mathematical structure of the nonlinear neuron dynamics, a circuit that balances both has been devised^36,37^. Recently, circuits have also been proposed that reduce energy consumption to the utmost limit by lowering the supply voltage to a few hundred mV or using an extremely small tunneling current^23,24,38^. These neuron circuits have been used for machine learning applications^3^, and also for optic, auditory, or other sensory signal processing^39–41^.

In this study, we fabricated a spiking neuron circuit, which is specially designed for waiting time generation in ultra-low power consumption. For the purpose of waiting time generation, the spiking neuron circuit implements the integrate-fire function while all the other biological functions were excluded intentionally. It is also designed to generate a nanosecond-width square pulse wave as the output spike for seamless connection with CMOS logic circuits with a common 1 V supply. The fabricated spiking neuron circuit consists of two parts: one part generates waiting time, and the other part generates a spike (Fig. 3b). In the former part, the input current is created by the ON current or subthreshold current of the transistor under the application of 1 V, and charges the capacitor with the approximately constant current. Then, after a waiting time that is determined by the ratio of the capacitance to the current, the capacitor potential (*V*_1_) reaches the threshold voltage of the inverter (around 0.5 V) and activates the spike generation part as shown in Fig. 3c. The capacitance was designed to be as small as possible to suppress power consumption, and metal–oxide–semiconductor (MOS) capacitors of several tens of fF or even smaller capacitance which is parasitic to transistors and wiring were used. The spike generation part consists of CMOS circuit. When the *V*_1_ reaches a threshold, positive feedback and delayed feedback are activated in turn to generate a spike output. Generally speaking, to reduce the power consumption of integrate–fire neuron circuits, methods such as lowering the supply voltage or using capacitive feedback have been used^23,24,42^, but here, in order to use a 1 V supply and reduce the use of capacitors as much as possible, we used only CMOS circuits.

**Figure 4:**
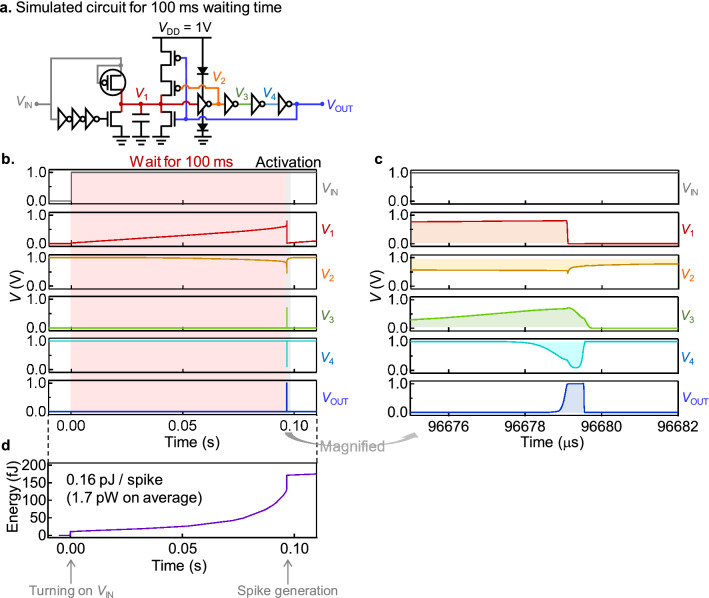
Simulation of the spiking neuron circuit. (**a**) The simulated spiking neuron circuit for the 100 ms waiting time. (**b**) The voltage wave forms at several different nodes in (**a**). The red shadowed region indicates the waiting time of this neuron circuit. (**c**) The magnification of (**b**) in the vicinity of the spike generation event, which is indicated by the grey shadow. (**d**) The simulated energy consumption of the spiking neuron circuit, which rapidly increases as approaching the spiking event due to the increase in the through current at the first-stage inverter. The detailed operation of the 1 μs spiking neuron circuit is also presented in Supplementary Note 4, and the simulations of all the spiking neuron circuits with different waiting times are presented in Supplementary Note 5.

To elucidate a detailed operation of the spiking neuron circuit, simulations are shown for the spiking neuron circuit that generates a waiting time of approximately 100 ms as shown in Fig. 4a. Transistor with 5 V withstand voltage in the TSMC 0.18 μm process was used (see Supplementary Notes 2 and 3 for more details) because the 5 V transistor under a 1 V supply leads to near-threshold computing and dramatically reduce power consumption. As shown in Fig. 4b, when 1 V is applied to *V*_in_ (input voltage in gray color), the *V*_1_ gradually increases (red color), and the spike voltage is output as *V*_out_ (blue color). Here, after the *V*_1_ reaching the threshold potential, the positive feedback raises *V*_1_ close to 1 V (red color), which contributes to the steep rise of the *V*_out_. Then, by resetting *V*_1_ to 0 V with delayed feedback, the *V*_out_ falls steeply and the spike waveform is completed. An enlarged view of the firing process (Fig. 4c) shows that the rise or the fall of the waveform becomes steeper with each successive inverter, in the order of *V*_1_, *V*_2_, *V*_3_, *V*_4_, and *V*_out_. Thus, by connecting CMOS inverters in multiple stages, a steep waveform can be obtained at the output side in a digitally compatible level, no matter how long the waiting time is at the input side. The energy consumption for a series of operations is sufficiently small, only 0.16 pJ per spike operation (Fig. 4d). This corresponds to an average power consumption of 1.7 pW, which is more than one order of magnitude lower than previous experimental demonstrations^20–24^. Here, to suppress energy consumption, diodes were inserted at the top and bottom of the first stage inverter (Fig. 4a), otherwise, the through current flows for a long time as the *V*_1_ approaches the inverter threshold and increases energy consumption. Simulation of a spiking neuron circuit with a shorter waiting time (approximately 1 μs) is also presented in Supplementary Note 4 and the simulations for all the other spiking neuron circuits are also summarized in Supplementary Note 5.

### Experiments on spiking neuron circuits

**Figure 5:**
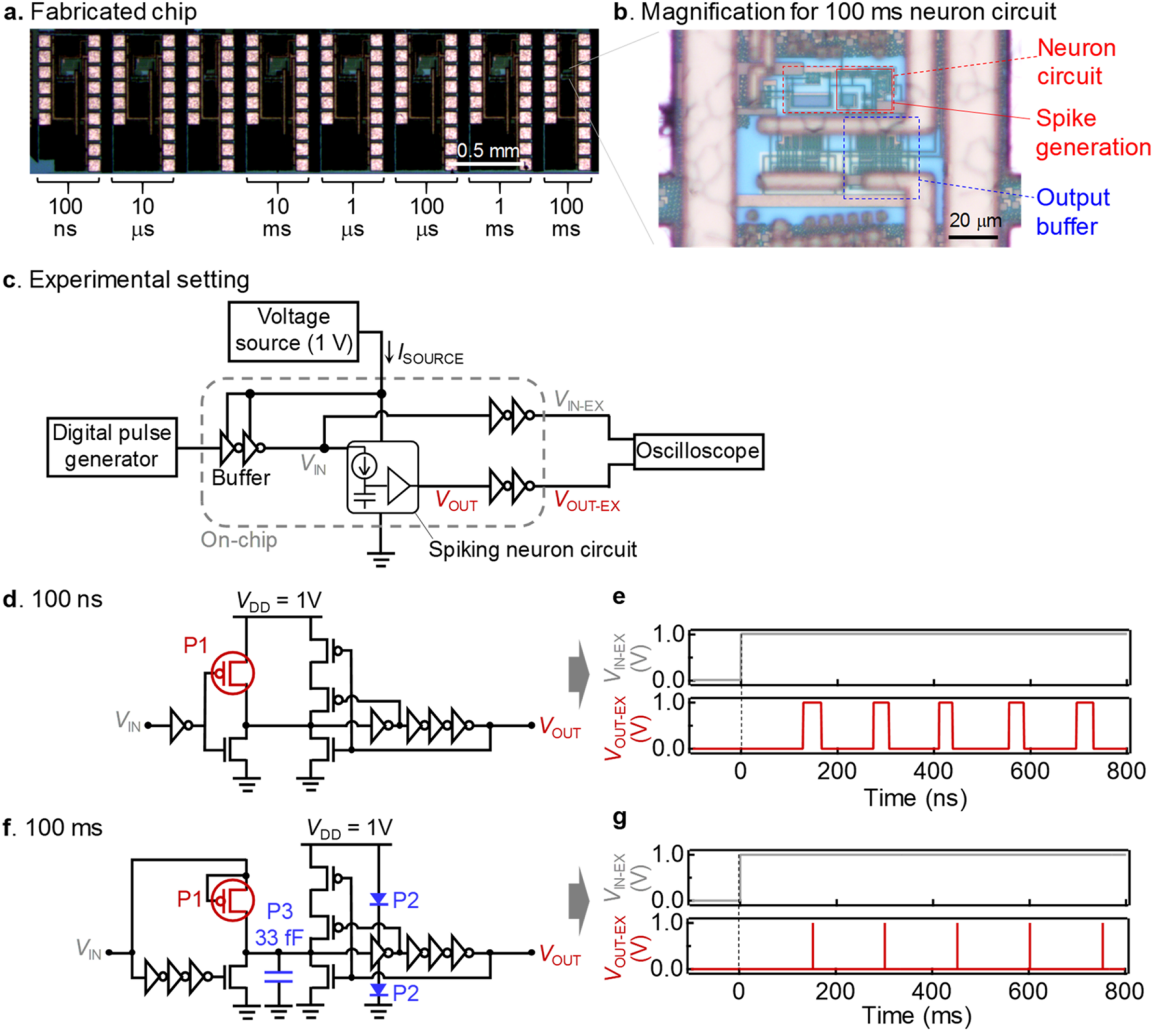
Experiments of the spiking neuron circuit. (**a**) An optical micrograph of the fabricated chip for spiking neuron circuits with various waiting times between 100 ns and 100 ms. (**b**) The magnification of the chip region for the spiking neuron circuit with the 100 ms waiting time. The red dashed square indicates the whole spiking neuron circuit while the red solid square corresponds to the spike generation part and the membrane capacitor. (**c**) The experimental setting for the measurement of the spiking neuron circuits. *V*_IN_ and *V*_OUT_ are the voltages inside the I/O buffers, and *V*_IN-EX_ and *V*_OUT-EX_ are the voltages outside the I/O buffers, which were measured by the oscilloscope. (**d**) A circuit diagram of the fabricated spiking neuron circuit for the 100 ns waiting time (see Supplementary Note 3 for details). (**e**) Experimentally measured wave forms of *V*_IN-EX_ and *V*_OUT-EX_ for the 100 ns spiking neuron circuit in (**d**). The interval between spikes are approximately 100 ns. (**f**) A circuit diagram of the fabricated spiking neuron circuit for the 100 ms waiting time (see Supplementary Note 3 for details). (**g**) Experimentally measured wave forms of *V*_IN-EX_ and *V*_OUT-EX_ for the 100 ms spiking neuron circuit in (**f**). The interval between spikes are approximately 100 ms. The experiments of all the spiking neuron circuits with different waiting times are presented in Supplementary Note 5.

Based on the simulation, the spiking neuron circuits were experimentally fabricated using the TSMC 180 nm BCD process as shown in Fig. 5a,b. By controlling the input current of the spiking neuron circuit (Supplementary Note 3), different lengths of waiting time can be generated from an input voltage of 1 V, which were measured based on the experimental setting as shown in Fig. 5c. In Fig. 5d,e, we used the ON current of the PMOS transistor to generate a waiting time in the order of 100 ns. Here, since the ON current of the PMOS can be tuned down to 10 nA with the length and width of the channel, the waiting time as short as 100 ns can be generated with a parasitic capacitance of a few fF. In Fig. 5f,g, instead, the sub-pA OFF current of the lower-threshold PMOS transistor with the 2 V withstand voltage was used to generate a waiting time in the order of 100 ms. In this case, an additional MOS capacitor of 33 fF was also utilized to elongate the waiting time. In this way, we succeeded in experimentally generating an arbitrary waiting time spanning six orders of magnitude from 100 ns to 100 ms on the chip. The circuits and the device parameters for all the other waiting times are also summarized in Supplementary Note 3.

**Figure 6:**
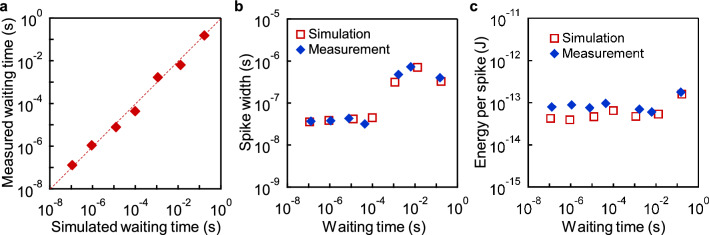
Comparison between experiments and simulations for spiking neuron circuits. (**a**) The experimentally measured waiting times of the fabricated spiking neuron circuits are plotted as a function of the simulating waiting times (post-layout simulation). The plot indicates the experimental results are approximately consistent with the simulation. (**b**) Experimentally obtained spike width and the simulated spike width as a function of the waiting time. (**c**) Experimentally obtained energy consumption per spike and the simulated energy consumption per spike as a function of the waiting time. In the experiment, the energy consumption was obtained by measuring *I*_SOURCE_ in Fig. 5c with Keithley 6430.

The waiting time in the simulation and the one obtained in the experiment were in general agreement throughout the six-order range of the waiting time (Fig. 6a and Supplementary Note 5). As shown in Fig. 6b, the output spike width was approximately 40 ns for the waiting time up to 100 μs, and approximately 400 ns for the longer waiting times due to the insertion of diodes at the first stage inverter as mentioned previously in Fig. 4a. This spike width can be converted to approximately 40 ns by using a spike width conversion circuit as shown in Supplementary Note 6. The steep rise and fall of the output spike guarantee the spike is compatible with CMOS logic circuits. The experimentally measured energy consumption per spike operation was found to be between 60 and 120 fJ for all the waiting times (Fig. 6c). It is interesting to note that the past examples of a neuron circuit with a long spike interval in the order of 100 ms is limited, and our 100 ms neuron circuit has the lowest power consumption of 1.2 pW among all the experimental demonstrations (Table 1). A comparison in Table 1 clearly shows the fabricated spiking neuron circuits have a unique feature of digital-circuit compatibility in the sense of the spike width and the supply voltage, and at the same time, a reasonably low energy consumption and a wide control range of the waiting time. It should be emphasized that all of these features are optimized for the waiting time generation inside a digital circuit and asynchronously controlling the switching circuits, rather than for the implementation of the biological functions or for the simple reduction of energy consumption in a single neuron circuit as in the case of previous studies^21,23,24^.

**Table 1:** Benchmark of neuron circuits.

References	Voltage (V)	Spike width (µs)	Digital circuit compatibility	Frequency (Hz)	Energy per spike (pJ)	Power (pW)	Process (nm)	Area (µm^2^)	Function
20	2.25	100	No	100	17	1700	350	1187	Spike frequency adaptation, positive feedback, refractory period
21	0.6	2600	No	100	0.4	40	90	442	Integrate-fire
22	1.8	–	Yes	8	2.8	22.4	180	116	Adaptive exponential I&F
23	0.2	17	No	26,000	0.004	105	65	35	Morris-Lecar
24	0.2	5000	No	16,000	0.002	30	65	31	Axon-Hillock
This work	1.0	0.4	Yes	6.6	0.18	1.2	180	504 (906)	Integrate-fire
This work	1.0	0.037	Yes	7,800,000	0.079	6,160,000	180	234 (401)	Integrate-fire

The characteristics of the spiking neuron circuit in this work are compared with the state-of-the-art neuron circuits that were experimentally fabricated in the past literature. The area only includes the membrane capacitor and the subsequent circuits while the values in the brackets include the current generation MOSFETs at the input of the spiking neuron circuits.

## Conclusion

In this study, a binary pattern generator in analogy to the biological central pattern generator was first applied to controlling the switching circuits for IoT devices. We utilized spiking neuron circuits to generate an arbitrary waiting time from 100 ns to 100 ms, and constructed a binary pattern generator for a switching-circuit control with ultra-low power. The generated binary waveform with an arbitrary temporal pattern was used to drive the gates of the switching transistors, and it is shown that the DC-DC voltage conversion circuit can be controlled with only several nanowatt. This is due to an extremely low power consumption of the spiking neuron circuits, as small as 1.2 pW at minimum which is the lowest ever among experimentally demonstrated neuron circuits. The binary pattern generator allows various control functions to be implemented without worrying about the power overhead of the control circuit in self-powered devices. In particular, energy harvesting circuits often involve various types of switching operations^25,43^, which could be simply implemented by the binary pattern generator. Low-power sensing could also exploit the advantage of the binary pattern generator; for example, the self-heating gas sensors require fast switching operations to minimize heating power^44^. Finally, it may also be useful for low-power wireless communication especially the one based on the pulse signals, where the intermittent pulse generation is the key to achieve extreme low power on average in the order of nanowatt^45,46^.

## Supplementary Information

Supplementary Information.

## Data Availability

The data that support the findings of this study are available from the authors on reasonable request.

## References

[1] Furber S, Temple S (2007). Neural systems engineering. J. R. Soc. Interface.

[2] Benjamin BV (2014). Neurogrid: A mixed-analog-digital multichip system for large-scale neural simulations. Proc. IEEE.

[3] Merolla PA (2014). A million spiking-neuron integrated circuit with a scalable communication network and interface. Science.

[4] Indiveri G, Liu S-C (2015). Memory and information processing in neuromorphic systems. Proc. IEEE.

[5] Ambrogio S (2018). Equivalent-accuracy accelerated neural network training using analogue memory. Nature.

[6] Li C (2018). Analogue signal and image processing with large memristor crossbars. Nat. Electron..

[7] Pei J (2019). Towards artificial general intelligence with hybrid Tianjic chip architecture. Nature.

[8] Wang Z (2019). Reinforcement learning with analogue memristor arrays. Nat. Electron..

[9] Marder E, Bucher D (2001). Central pattern generators and the control of rhythmic movements. Curr. Biol..

[10] Ijspeert AJ (2008). Central pattern generators for locomotion control in animals and robots: A review. Neural Netw..

[11] Taga G, Yamaguchi Y, Shimizu H (1991). Self-organized control of bipedal locomotion by neural oscillators in unpredictable environment. Biol. Cybern..

[12] Jean A (2001). Brain stem control of swallowing: Neuronal network and cellular mechanisms. Physiol. Rev..

[13] Tilden, M. W. Adaptive robotic nervous systems and control circuits therefore. *U.S. Patent* 5325031 (1994).

[14] Hrynkiw DM, Tilden MW (2002). Junkbots, Bugbots, and Bots on Wheels.

[15] Lee YJ (2007). Low power CMOS electronic central pattern generator design for a biomimetic underwater robot. Neurocomputing.

[16] Hotta Y (2008). Cooperative dynamics of an artificial stochastic resonant system. Appl. Phys. Exp..

[17] Maruyama A, Ichimura T, Maeda Y (2015). Hard-wired central-pattern generator hardware network for quadrupedal locomotion based on neuron and synapse models. Adv. Biomed. Eng..

[18] Vogelstein RJ, Tenore FVG, Guevremont L, Etienne-Cummings R, Mushahwar VK (2020). A silicon central pattern generator controls locomotion in vivo. IEEE Trans. Biomed. Circ. Syst..

[19] Donati E, Krause R, Indiveri G (2021). Neuromorphic pattern generation circuits for bioelectronic medicine. Int. IEEE/EMBS Conf. Neural Eng..

[20] Basu A, Hasler PE (2010). Nullcline-based-design of a silicon neuron. IEEE Trans. Circ. Syst..

[21] Cruz-Albrecht JM, Yung MW, Srinivasa N (2012). Energy-efficient neuron, synapse and STDP integrated circuits. IEEE Trans. Biomed. Circ. Syst..

[22] Indiveri G, Corradi F, Qiao N (2015). Neuromorphic architectures for spiking deep neural networks. IEDM.

[23] Sourikopoulos I (2017). A 4-fJ/spike artificial neuron in 65 nm CMOS technology. Front. Neurosci..

[24] Danneville F (2019). A sub-35 pW axon-hillock artificial neuron circuit. Solid State Electron..

[25] Lefeuvre E, Audigier D, Richard C, Guyomar D (2007). Buck-boost converter for sensorless power optimization of piezoelectric energy harvester. IEEE Trans. Power Electron..

[26] Hodgkin L, Katz B (1949). The effect of sodium ions on the electrical activity of the giant axon of the squid. J. Physiol..

[27] Hodgkin H, Huxley AF (1952). A quantitative description of membrane current and its application to conduction and excitation in nerve. J. Physiol..

[28] Indiveri G (2011). Neuromorphic silicon neuron circuits. Front. Neurosi..

[29] Hynna KM, Boahen K (2007). Thermodynamically equivalent silicon models of voltage-dependent ion channels. Neural Comput..

[30] Qiao N, Indiveri G (2016). Scaling mixed-signal neuromorphic processors to 28 nm FD-SOI technologies. IEEE BioCAS.

[31] Tohara T (2016). Silicon nanodisk array with a fin field-effect transistor for time-domain weighted sum calculation toward massively parallel spiking neural networks. Appl. Phys. Exp..

[32] Kornijcuk V (2016). Leaky integrate-and-fire neuron circuit based on floating-gate integrator. Front. Neurosci..

[33] Izhikevich EM (2003). Simple model of spiking neurons. IEEE Trans. Neural. Netw..

[34] Pickett MD, Medeiros-Ribeiro G, Williams RS (2013). A scalable neuristor built with Mott memristors. Nature Mater..

[35] Tuma T (2016). Stochastic phase-change neurons. Nat. Nanotechnol..

[36] Wijekoon JHB, Dudek P (2008). Compact silicon neuron circuit with spiking and bursting behavior. Neural Netw..

[37] Kohno T, Sekikawa M, Li J, Nanami T, Aihara K (2016). Qualitative-modeling-based silicon neurons and their networks. Front. Neurosci..

[38] Chavan T, Datta S, Mohapatra NR, Ganguly U (2020). Band-to-band tunneling based-ultra-energy-efficient silicon neuron. IEEE Trans. Electron Devices.

[39] Osswalt M, Ieng S-H, Benosman R, Indiveri G (2017). A spiking neural network model of 3D perception for event-based neuromorphic stereo vision systems. Sci. Rep..

[40] Stoliar P, Schneegans O, Rozenberg MJ (2021). A functional spiking neural network of ultra compact neurons. Front. Neurosci..

[41] Haessig G (2020). Event-based computation for touch localization based on precise spike timing. Front. Neuronsci..

[42] Mead C (1989). Analog VLSI and Neural Systems.

[43] Guyomar D, Lallart M (2011). Recent progress in piezoelectric conversion and energy harvesting using nonlinear electronic interfaces and issues in small scale implementation. Micromachines.

[44] Tanaka T (2019). Low-power and ppm-level multimolecule detection by integration of self-heated metal nanosheet sensors. IEEE Trans. Electron Dev..

[45] Morimura H (2014). Ultra-low-power circuit techniques for mm-size wireless sensor nodes with energy harvesting. IEICE Electron. Exp..

[46] Niitsu K (2018). Energy-autonomous biosensing platform using supply-sensing CMOS integrated sensor and biofuel cell for next-generation healthcare Internet of Things. Jpn. J. Appl. Phys..

