# Supplementary material for: Ultra-low-power switching circuits based on a binary pattern generator with spiking neurons
Source: Sci Rep. 2022 Jan 21;12:1150. doi: 10.1038/s41598-022-04982-w (PMC8782828; doi:10.1038/s41598-022-04982-w)
Supplement: Supplementary file 1 — Supplementary Information. [file 41598_2022_4982_MOESM1_ESM.docx]

**(Supplementary Information)**

**Ultra-low-power switching circuits based on a binary pattern generator with spiking neurons**

Takeaki Yajima^1*^

^1^Department of Electronics, Kyushu University, Fukuoka-shi, Fukuoka 819-0395, Japan

^*^Correspondence to yajima@ed.kyushu-u.ac.jp

**Supplementary Note 1: Benchmark of DC-DC converters**

The efficiency of the DC-DC converter in this study is compared with previous studies in Fig. S1, especially focusing on the low output power region. While most of the previous demonstrations show rapid degradation below 1 × 10^-6^ W output power, the converter in this study maintains efficiency around 90 % down to 1 × 10^-8^ W.

**Supplementary Fig. S1:** **Benchmark of DCDC converters.** Efficiency vs. output power is summarized for buck converters and boost converters in the past studies. The buck-boost converter in this study is plotted in red. The plot especially focuses on the low output power region below 1 × 10^-5^ W.

**Supplementary Note 2: Transfer characteristics of transistors**

All the NMOS transistors in the spiking neuron circuits and the logic circuits have the 5 V withstand voltage and the channel length of 800 nm and the width of 400 nm in the TSMC 180 nm process unless stated. All the PMOS transistors have the 5 V withstand voltage and the channel length of 800 nm and the width of 600 nm unless stated. The transfer characteristics of these transistors at the drain voltage (*V*_D_) of 1 V are shown in Fig. S2.

**Supplementary Fig. S2:** **Transfer characteristics.** The simulated transfer characteristics of the NMOS and PMOS transistors that are used in the spiking neuron circuits and the logic circuits.

**Supplementary Note 3: Circuit diagrams of spiking neuron circuits.**

**Supplementary Table S1: Transistor parameters.**

**Supplementary Fig. S3: Circuit diagrams of spiking neuron circuits.**

Spiking neuron circuits for waiting times between 100 ns and 100 ms were experimentally fabricated. All the circuit diagrams of these spiking neuron circuits are shown in Fig. S3. In each diagram, P1, P2, and P3 indicate PMOS transistors, and N1 indicates an NMOS transistor. The red circle indicates the transistor that determines the input current to the spiking neuron circuit. For all the transistors, the typical withstand voltage (“process”), the channel length (*L*), and the channel width (*W*) are summarized in Table S1.

**Supplementary Note 4: Simulation of the 1 μs spiking neuron circuit.**

The simulated waveforms and energy consumption are shown for the 1 μs spiking neuron circuit in Fig. S4. When 1 V is applied to *V*_in_ (input voltage in gray color), the *V*_1_ gradually increases (red color), and the spike voltage is output as *V*_out_ (blue color) as shown in Fig. S4a. Here, after the *V*_1_ reaching the threshold potential, the positive feedback slightly raises *V*_1_ (red color), which contributes to the steep rise of the *V*_out_. Then, by resetting *V*_1_ to 0 V with delayed feedback, the *V*_out_ falls steeply and the spike waveform is completed. An enlarged view of the firing process (Fig. S4b) shows that the rise or the fall of the waveform becomes steeper with each successive inverter, in the order of *V*_1_, *V*_2_, *V*_3_, *V*_4_, and *V*_out_. The energy consumption for a series of operations is small, only 39 fJ per spike operation (Fig. S4c). The simulated circuit diagram and the definition of each voltage are shown in Fig. S4d.

**Supplementary Fig. S4:** **Simulation of the 1 μs spiking neuron circuit.** (**a**) Simulation results of the 1 μs spiking neuron circuit. (**b**) The enlarged view of the simulation results around the firing process. (**c**) The simulated energy consumption of the spiking neuron circuit. (**d**) The circuit diagram for the simulations in (**a-c**).

**Supplementary Note 5: Simulation and experiments on spiking neuron circuits.**

Waiting times in the range between 100 ns and 100 ms were generated by spiking neuron circuits shown in Fig. S3. Their results are shown in Fig. S5 (simulation) and in Fig. S6 (experiments). They show *V*_IN_ (*V*_IN-EX_) and *V*_OUT_ (*V*_OUT-EX_) in the spiking neuron circuits as a function of time.

**Supplementary Fig. S5: Simulation of spiking neuron circuits.** Simulation results for the spiking neuron circuits with various different waiting times (post-layout simulation).

**Supplementary Fig. S6: Experiments of spiking neuron circuits.** Experimental results for the spiking neuron circuits with various different waiting times. The wave forms were measured in the measurement system of Fig. 5c.

**Supplementary Note 6: Spike width conversion**

Spiking neuron circuits for waiting times above 1 ms show longer spike width (around 400 ns) than those below 100 μs (around 40 ns). This is because the spiking neuron circuits for longer waiting times have extra diodes above and below the first-stage inverter to save energy, and these extra diodes slow down the spike generation part of the spiking neuron circuits. In order to recover the short spike width of around 40 ns, a pulse-to-spike converter circuit as shown in Fig. S7 is inserted after the spiking neuron circuits above 1 ms.

The pulse-to-spike converter consists of two 100 ns spiking neuron circuits, a latch circuit, and other logic circuits (Fig. S7a). It receives the input pulse voltage (*V*_IN_) and outputs two spike voltages: one at the rise of *V*_IN_ (*V*_UPSPK_), and the other at the fall of *V*_IN_ (*V*_DNSPK_). The simulated waveforms for a 1 μs-width input pulse are shown in Fig. S7b. Then, this pulse-to-spike converter circuit was inserted after the 100 ms spiking neuron circuit as shown in Fig. S7c. The simulated results for these circuits are shown in Figs. S7d and S7e, where the long spike width of the 100 ms spiking neuron circuit (*V*_OUT1_) is successfully converted to the short spike width (*V*_OUT2_).

**Supplementary Fig. S7:** **Spike width conversion.** (**a**) A circuit diagram of the pulse-to-spike converter circuit. (**b**) The simulated waveforms for (a). (**c**) The combination of a 100 ms spiking neuron circuit and the pules-to-spike converter circuit. (**d**) The simulated waveforms for (c). (**e**) An enlarged view of (d).

**Reference**

1. Adami, S.-E. et al. Self-powered ultra-low power DC-DC converter for RF energy harvesting. *IEEE Faible Tension Faible Consommation* (2012).
2. Sarkar, S. et al. Design of an ultra-low powered DC-DC buck converter for wireless sensor networks. *Asia Pacific Conference on Postgraduate Research in Microelectronics & Electronics,* 126-131 (2012).
3. Bouler, Ⅲ, D. W., Baxter, J. & Costinett, D. Optimization of GaN-based ultra-low power boost converter in far-field energy harvesting. *IEEE PELS Workshop on Emerging Technologies: Wireless Power Transfer* (2016).
4. Fan, S. et al. An ultra-low power (ULP) zero-current detector (ZCD) circuit for switching inductor converter applied in energy harvesting system. *IEEE Electrical Design of Advanced Packaging and Systems Symposium*, 1-3 (2017).
5. Santoro, F. et al. A 92.1% efficient DC-DC converter for ultra-low power microcontrollers with fast wake-up. *IEEE Custom Integrated Circuits Conference* (2017).
6. Asano, H. et al. A fully integrated, wide-load-range, high-power-conversion-efficiency switched capacitor DC–DC converter with adaptive bias comparator for ultra-low-power power management integrated circuit. *Jpn. J. Appl. Phys.* **57**, 04FF03 (2018).
